# Validation of the Portuguese DSM-IV-MR-J

**DOI:** 10.1007/s11469-016-9708-9

**Published:** 2016-10-28

**Authors:** Filipa Calado, Joana Alexandre, Mark D. Griffiths

**Affiliations:** 1 International Gaming Research Unit, Psychology Division, Nottingham Trent University, 50 Shakespeare Street, NG1 4FQ Nottingham, UK; 2 CIS/ISCTE- Lisbon University Institute, Avenida das Forcas Armadas, 1649-026 Lisbon, Portugal

**Keywords:** DSM-IV-MR-J, Problem gambling, Screening, Psychometric properties, Youth, Portuguese samples

## Abstract

Youth problem gambling is viewed as an emergent public health issue in many countries, and is also an emerging area of public concern in Portugal. However, there is currently no Portuguese instrument that focuses specifically on the measurement of problem gambling among young people. Consequently, the present study aimed to validate the DSM-IV-MR-J for use among Portuguese adolescents and to examine its’ psychometric properties. A cross-cultural adaption of this instrument to the Portuguese language was performed using the translation and back translation method. The final version of the instrument was administered to 753 Portuguese high school and first year college students. The findings revealed an acceptable internal reliability and replicated the one-factor structure of this scale. Based on these findings, the Portuguese DSM-IV-MR-J appears to be a valid and reliable instrument, and provides a much needed psychometric tool for the development of more research on youth gambling in Portugal.

The empirical literature indicates that adolescents are highly vulnerable to gambling (Chambers & Potenza, 2003; Griffiths, 2011) and, at present, gambling is seen as an emergent area of interest both within the fields of adolescent risk behavior and gambling studies. Despite age prohibitions in many countries all around the world to protect minors from gambling, most empirical research demonstrates that a large proportion of adolescents engage in gambling, with a rate of problem gambling significantly higher than that found in adults (Molinaro et al., 2014). Furthermore, the current generation of youth has grown up in an era where gambling opportunities are widespread and socially acceptable (Volberg et al. 2010; Gupta & Derevensky, 2000).

In addition, the development of technology has generated new forms of gambling via the Internet, mobile phone and interactive television (Griffiths & Parke, 2010). It has also been argued that youth are more receptive to modern forms of gambling than their predecessors because of the apparent similarity between these games and other familiar technology-based games (Delfabbro, King, Lambos, & Puglies, 2009). Therefore, given this widespread availability of gambling opportunities, which puts more young people at risk of developing gambling-related problems, there is a growing need to assess problem gambling among adolescents and young adults using the most robust psychometric instruments. In the adolescent gambling field, one of the most widely used instruments to assess problem gambling among this age group has been the DSM-IV-MR-J (i.e., the juvenile multiple-response version of the DSM-IV criteria for pathological gambling; Fisher, 2000). This instrument has been administered in a significant number of countries (e.g., Great Britain, Canada, Iceland), and there are several studies conducted in different cultural contexts (e.g., Lithuania, Finland), which showed that its psychometric properties are acceptable. However, despite this increase in assessing youth problem gambling in many countries across the world, currently there is no instrument to assess problem gambling among this age group in Portugal.

At present, the Portuguese gambling market comprises lotteries (“*Lotaria Clássica*”, “*Lotaria Popular*”, “*Lotaria Instantânea*”, “*Totoloto*”, “*Totobola*”), which are owned by the State, as well as casino games, television quizzes, and Internet games (Lopes 2013). Until recently, online gambling was legally prohibited, but the Government legalized online gambling in April 2015 [Decreto Lei no 64/2015], in an attempt to generate new sources of income to finance the increasing budget deficits the country currently faces. According to the new Act, the Government will provide licenses, without any kind of exclusivity, to gambling companies that want to operate within Portugal [Decreto Lei no 64/2015].

Research in the gambling field is very scarce in Portugal and there is little published in peer-reviewed journals. However, in the last few years, some studies have been conducted with the aim of increasing the scientific knowledge of this phenomenon, but are only available in Portuguese. For instance, a study conducted by Lopes (2009) with a sample of 3,850 individuals aged 18–70 years found a problem gambling rate of 0.2 % using the South Oaks Gambling Screen (SOGS). This study highlighted that gambling was prevalent among older-aged youth, although there was still a great knowledge gap about gambling among this age group and younger teenagers (Lopes, 2009). Another study conducted by Hubert (2015) found that 17 % of online problem gamblers (as measured by the SOGS) were aged between 16 and 20 years.

Although Portugal has an age limit of 18 years to engage in any legal commercial gambling activity, recent news reports about the involvement of underage youth gambling, more specifically on the sports betting game *Placard*, have recently emerged (Spranger 2016). In addition, a small-scale qualitative study conducted among the Portuguese youth, revealed that underage youth engage in gambling, and showed a positive perception towards the activity (Calado et al. 2014), emphasizing the need for a psychometric instrument that assesses youth problem gambling in the Portuguese cultural context. Given that there is currently no Portuguese instrument specifically assessing problem gambling among young people, the goal of the present study was to adapt to the DSM-IV-MR-J for use in the Portuguese context.

## Method

### Sample

**Fig. 1:**
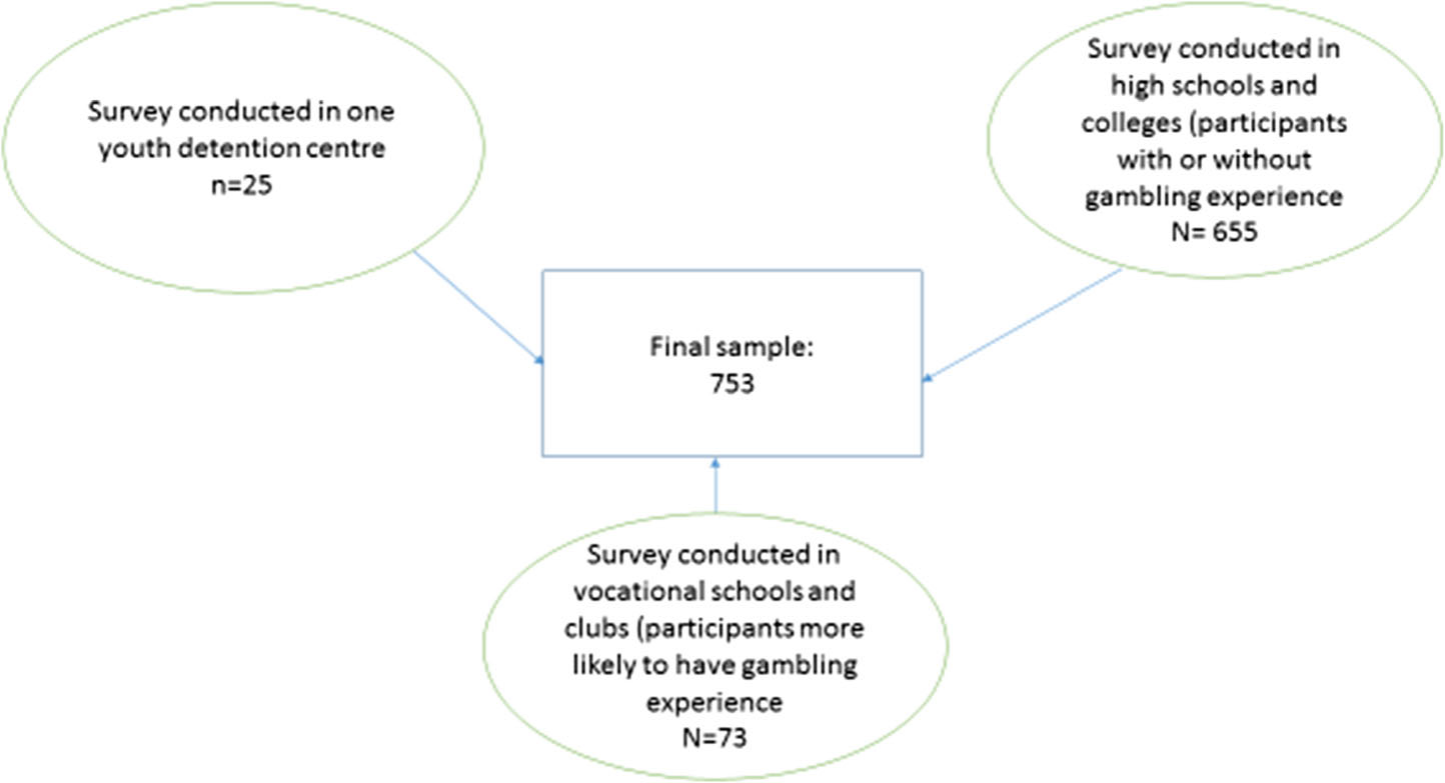
Sampling procedure employed in the present study

The participants comprised 753 adolescents and young adults (65.5 % males, 34.5 % females; mean age = 18.9 years, *SD =* 2.6) attending high schools and first years of college in Portugal. Although the sample was not representative of all Portuguese youth, an effort to collect data from several regions of the country was made by the authors (i.e., Lisbon, Oporto and Alentejo). In addition, data were collected from adolescents (i) with and without gambling experience attending regular high schools, (ii) attending vocational schools where gambling was thought to be more likely, and (iii) in one youth detention center where gambling was thought to be highly likely. This oversampling procedure has already been used in validations of other gambling instruments (e.g., Hayer, 2014) and had the main aim of creating a higher variance of gambling in the sample in order to better analyze the psychometric properties of the scale. Due to the fact that adolescent problem gambling is a low prevalence phenomenon among the general population, the authors tried to oversample individuals from risk segments with possible gambling experiences to understand the most important items in the factorial structure of this instrument in a new cultural context. This sampling procedure is described in further detail in Fig. 1.

### Measures

#### Sociodemographics, Gambling Frequency and Money Spent on Gambling

Demographic data were collected on age and gender. Participants were also asked to indicate how often they had gambled during the past year from 1 (“never”) to 6 (“everyday”), as well as the money they had spent on any gambling activity during the previous year from 1 (“had never bet money on gambling”) to 5 (“had spent up to 1000 Euros”).

#### DSM-IV-Multiple Response-Juvenile (DSM-IV-MR-J)

DSM-IV-MR-J is a psychometrically validated tool developed by Fisher (2000) for assessing adolescent problem gambling among those who had gambled during the past year. This instrument contains nine items, and assesses a number of important variables related to youth problem gambling, such as progression and preoccupation, tolerance, withdrawal and loss of control, escape, chasing, lies and deception, illegal activities, and family and school disruption. The response categories comprise 1 = “never”, 2 = “once or twice”, 3 = “sometimes” and 4 = “often”. Scoring of criteria 1: often = 1 point, criteria 2–6: sometimes and often = 1 point, and criteria 7–9: once or twice, sometimes and often = 1 point Total score (range 0–9) was calculated by summing up the scores of all items. Participants who obtain a score of 0 or 1 are classified as social gamblers, a score of 2 or 3 indicates at-risk gambling, and a score of 4 or more indicates problem gambling. The DSM-IV-MR-J instrument has been shown to be reliable and valid among young people. In the original version of the DSM-IV-MR-J, the Cronbach’s alpha value was 0.75 (Fisher, 2000).

#### Psychosocial Measures

A range of psychosocial measures were also administered in order to assess criterion validity, and included: the Portuguese version of Depression Anxiety and Stress Scale–21 (DASS-21) (Pais-Ribeiro et al., 2004), a 21-item scale comprising three 7-item subscales rated on a 4-point scale (i.e., 0 = “Did not apply to me at all” to 3 = “Applied to me very much”, or “most of the time”) to assess symptomatology of depression, stress, and anxiety, with Cronbach’ alphas of 0.89 (Depression), 0.84 (Anxiety), and 0.88 (Stress) in the present study; The Portuguese version of the Rosenberg Self Esteem Scale (Pechorro et al., 2011), a 10-item scale with items scored 1 = “Strongly agree” to 4 = “Strongly Disagree”, with a Cronbach’s alpha of 0.89 in the present study; and the Portuguese version of the Adapted Self-Report Delinquency Scale (ASRDS) (Pechorro et al., 2015), a 35-item scale that assesses self-reported delinquent and antisocial behaviors in youths, rated on a 3-point scale (i.e., 1 = “Never” to 3=”Often”), with a Cronbach’s alpha of 0.89 in the present study.

### Procedure

Translation and back translation of the DSM-IV-MR-J was performed using the following steps (Beaton et al., 2000): (1) Initial translations from the English language version to the target language (i.e., Portuguese) by the first and second authors, Portuguese native speakers; (2) synthesis of the translations to resolve discrepancies between both translators (3) backward translations of the new target language to English by another translator fluent in Portuguese, who worked independently of stage one; and (4) review of translations to reach consensus and produce the final version. The final version of the Portuguese DSM-IV-MR-J is in the Appendix.

For recruiting adolescents, an information letter explaining the purpose of the present study was sent to the headmaster of four high schools, two vocational schools, one youth club, and one youth detention center. If the headmaster provided permission, another letter was sent to students and their parents (if participants were minors). Only participants who provided their full informed consent participated in the study. For recruiting young adults, an information letter was sent to three public universities in Portugal by the authors. As with adolescents, only participants who provided their full informed consent participated in this research.

Students completed the survey individually during class time, and were instructed that their participation was completely voluntary. Participants were requested not to write their names in order to maintain anonymity. Finally, the students were offered the possibility of contact with the authors in case they had questions or concerns regarding the study. The institutional review committee of the research team’s university ethics committee provided approval for the study.

### Analytic Strategy and Statistical Analyses

Statistical analysis comprised (i) descriptive analysis for ascertaining the prevalence of problem gambling and the most frequent gambling activities; (ii) assessment of internal reliability using Cronbach’s alpha coefficient; (iii) assessment of the criterion validity of the DSM-IV-MR-J by examining its correlation with gambling frequency and the psychosocial measures of depression, anxiety, stress, self-esteem, and delinquency; and (iv) assessment of the construct validity of the DSM-IV-J-MR by means of a confirmatory factor analysis (CFA). All statistical analyses were performed using Mplus 7.2 and IBM SPSS Statistics 22.

## Results

### Descriptive Statistics

A total of 43.2 % of participants reported they had gambled less than once a month, 9.1 % reported they had gambled once a month, and 14.4 % reported they had gambled at least once per week. Based on the DSM-IV-MR-J, 3.5 % of participants were categorized as problem gamblers, with a further 9 % classified as at-risk gamblers, 53.5 % as social gamblers, and 33.3 % as non-gamblers. The most frequent gambling activities reported by participants were sports betting (14.9 %), scratch cards (14.9 %) and lottery games (13.6 %). When questioned about online gambling, the most reported gambling games were sports betting (10.6 %) and playing the ‘free play’/’demo’ mode on internet gambling sites (7.8 %).

### Internal Reliability

The reliability of the DSM-IV-MR-J as assessed by the Cronbach’s alpha was 0.72 and could not be improved upon deletion of any item. A score of 0.70 or greater is generally considered to be acceptable (Blacker & Endicott, 2002), especially in scales of this size.

### Criterion Validity

Criterion validity is a comparison of the measure with external validators that are likely to be associated with problem gambling. The DSM-IV-MR-J showed significant correlations with all measures used (see Table 1). More specifically, problem gambling positively correlated with depression, anxiety, stress, and delinquency, and negatively correlated with self-esteem. In addition, another way in which criterion validity can be assessed is to examine differences between problem, at-risk, and non-problem gamblers with respect to behaviors related to gambling difficulties but which are not included in DSM-IV-MR-J, such as the frequency of gambling, and the largest amount of money ever gambled in the past 12 months. Therefore, as observed in Table 2 (using chi-square tests), participants who scored as problem and at-risk gamblers are more likely to had gambled more frequently (*p* < 0.0001).

**Table 1:** Relationship between DSM-IV-MR-J and other psychosocial measures

	DSM-IV-MR-J
	r Spearman
Self-Report Delinquency	0.39**
Depression	0.20**
Anxiety	0.21**
Stress	0.23**
Self-esteem	−0.13**

*p < 0.05: **p < 0.01; NS Not Significant

**Table 2:** DSM-IV-MR-J according to gambling frequency

			DSM-IV-MR-J scores		
			No problem gambling	At-risk gambling	Problem gambling
Gambling Frequency	Never	N	247	0	0
		% of Total	33.2 %	0 %	0 %
	Less than once per month	N	302	21	0
		% of Total	40.6 %	2.8 %	0 %
	Once per month	N	49	14	4
		% of Total	6.6 %	1.9 %	0.5 %
	At least once per week	N	53	32	22
		% of Total	7.1 %	4.3 %	3 %

Table 3 indicates that people who scored as problem and at-risk gamblers were more likely to spend a larger amount of money in gambling (*p* < 0.0001).

**Table 3:** DSM-IV-MR-J scores according to the amount of money spent on gambling

			DSM-IV-MR-J scores		
			No problem gambling	At-risk gambling	Problem gambling
Amount of money spent on gambling	Never bet money on gambling	N	258	2	0
		% of Total	34.5 %	0.3 %	0 %
	Between less than 1 euro and 10 Euros	N	290	22	1
		% of Total	38.8 %	2.9 %	0.1 %
	Between 10 and 1000 Euros	N	105	44	25
		% of Total	14.1 %	5.9 %	3.3 %

### Construct Validity

Finally, a confirmatory factor analysis (CFA) was performed on the nine items of the instrument to test the previously established one-factor solution of the DSM-IV-MR-J (Fisher, 2000), through categorical weighted least squares confirmatory factor analysis implemented using Mplus software (Muthén & Muthén, 2004). Conventional fit indices, independent of the sample size, were used to examine the goodness of fit of the model under analysis: root mean square error of approximation (RMSEA), the comparative fit index (CFI) and the Tucker-Lewis index (TLI) (Vandenberg, 2006).

The results indicated that a single-factor model adequately represents the structure of the DSM-IV-MR-J. More specifically, the comparative fit index (CFI) and the Tucker-Lewis index (TLI) were .95 and .93, respectively, and the root mean square error of approximation (RMSEA) was .05, indicating a good fit (Hu & Bentler 1999; Loo et al., 2011; Brown, 2015). In addition, factor loadings were all significant (*p* < .001), ranging from .623 to .855 (see Table 4).

**Table 4:** Standardized factor loadings of the Portuguese DSM-IV-MR-J items

Item	Factor loading
1.In the past year, how often have you found yourself thinking about gambling or planning to gamble	0.647
2.During the course of the past year, have you needed to gamble with more and more money to get the amount of excitement you want?	0.722
*3.*In the past year, have you ever spent much more than you planned to on gambling?	0.623
4.In the past year, have you felt bad or fed up when trying to cut down or stop gambling?	0.724
*5.*In the past year, how often have you gambled to help you to escape from problems or when you are feeling bad?	0.752
6.In the past year, after losing money gambling, have you returned another day to try and win back money you lost?	0.730
*7.*In the past year, has your gambling ever led to: lies to your family?	0.850
*8.*In the past year, have you ever taken money from the following without permission to spend on gambling: school diner or fare money? Money from your family? Money from outside the family?	0.781
*9.*In the past year, has your gambling ever led to arguments with family/friends or others, or missing school?	0.847

## Discussion

The aim of the present study was to validate the Portuguese version of DSM-IV-MR-J and test its psychometric properties. In fact, this study is the first in Portugal to use a youth problem gambling instrument and to provide a prevalence rate of gambling and problem gambling among Portuguese youth. In Portugal, some concerns have been raised about the availability of legal gambling opportunities, especially since the legalization of online gambling in 2015, which may be associated with the prevalence of youth disordered gambling found in the present study. Therefore, it seems crucial to validate instruments to assess gambling-related problems among this age group in Portugal.

Internal consistency reliability of the Portuguese version of the DSM-IV-MR-J was acceptable, especially given the size of this instrument. In fact, the Cronbach’s alpha coefficient in the present study (0.72) was very similar to the 0.75 found by Fisher (2000) in the development of the original DSM-IV-MR-J. Moreover, the Cronbach’s alpha did not increase upon deletion of any item, and thus provides evidence for a solid internal consistency of the scale.

In addition, construct validation was conducted by means of a CFA. The results of the CFA provided support for the previously established unidimensionality of this instrument as the model fitted the data adequately. Moreover, it should be noted that all items of the DSM-IV-MR-J were statistically significant and relatively high (above the conventional threshold of 0.5), which lends further support to the construct validity of the Portuguese version of the DSM-IV-MR-J, and that all items can be interpreted as symptoms and/or consequences of problem gambling.

In addition, the Portuguese version of the DSM-IV-MR-J showed a statistically significant correlation with other relevant psychosocial variables, which have been found to be positively associated with problem gambling in the empirical literature, such as delinquency, depression, anxiety and stress (Ste-Marie et al. 2006; Sheela et al. 2015). Moreover, the DSM-IV-MR-J showed a significant negative correlation with self-esteem, which was in the expected direction and also in line with previous validations of other gambling instruments (e.g., Tolchard & Delfabbro, 2013) and with other studies (Delfabbro et al. 2006). Although the significant correlations were modest, they appear to lend support to the view that youth problem gamblers may show some clinically significant problems, due to the fact that they occurred in the expected direction. Furthermore, adolescents classified as problem and at-risk gamblers based on DSM-IV-MR-J scores, were significantly different from social gamblers in other aspects. More specifically, problem gamblers and those at-risk gambled more often, and they were significantly more likely than social gamblers to spend higher amounts of money on gambling. These findings are also in line with previous studies of the psychometric properties of the DSM-IV-MR-J in other cultural contexts (e.g., Skokauskas, Burba, & Freedman, 2009; Castren et al. 2015).

It is also worth noting that the present study also had some limitations, which should be kept in mind when interpreting the findings. Most importantly, the present study exclusively utilized self-report data, which are prone to various well known biases, such as social desirability and memory recall biases. Furthermore, the sample size was modest and not nationally representative of all Portuguese youth. However, the study did recruit from different segments of young people and in different regions of the country, which is an essential procedure for validating a scale in a new cultural context. Therefore, the present research provided an important contribution to the study of the psychometric properties of a widely used instrument to assess problem gambling among young people in a different country and cultural context. In addition, the present study showed that problem gambling appears to be an issue for some Portuguese youth. It is hoped that the validation of the DSM-IV-MR-J in the present study will encourage future research concerning youth gambling in Portugal, and will contribute for raising awareness of this phenomenon among Portuguese researchers, clinicians, schools, gaming industry, policymakers, and politicians.

## Appendix

(Table 5).

**Table 5:** Portuguese Version of DSM-IV-Multiple Response-Juvenile (DSM-IV-MR-J)

Items	Questions
Item 1	Durante o ano passado, quantas vezes te apercebeste de que estavas a pensar sobre jogo a dinheiro ou a planear jogar (a dinheiro)? Nunca/Uma ou duas vezes/Algumas vezes/Frequentemente
Item 2	Durante o ano passado, precisaste de jogar com maiores quantidades de dinheiro para conseguires obter o nível de excitação que querias? Sim/Não
Item 3	Durante o ano passado, alguma vez gastaste muito mais dinheiro do que aquele que tinhas pensado gastar em jogo a dinheiro? Nunca/Uma ou duas vezes/Algumas vezes/Frequentemente
Item 4	Durante o ano passado, sentiste-me mal contigo próprio(a) ou ficaste saturado(a) quando tentaste reduzir ou parar de jogar a dinheiro? Nunca/Uma ou duas vezes/Algumas vezes/Frequentemente
Item 5	Durante o ano passado, quantas vezes jogaste a dinheiro como forma de te ajudar a fugir dos teus problemas ou quando te sentiste mal? Nunca/Uma ou duas vezes/Algumas vezes/Frequentemente
Item 6	Durante o ano passado, depois de perderes dinheiro a jogar voltaste a jogar passado uns dias para voltar a recuperar o dinheiro que perdeste? Nunca/Menos de metade das vezes/Mais de metade das vezes/Sempre
Item 7	No ano passado, jogar a dinheiro levou-te a mentir à tua família? Nunca/Uma ou duas vezes/Algumas vezes/Frequentemente
Item 8	No ano passado, alguma vez tiraste dinheiro dos seguintes sem a devida autorização para gastares em jogo a dinheiro Dinheiro de refeições da escola? Dinheiro da tua família? Dinheiro de alguém de fora da tua família? Nunca/Uma ou duas vezes/Algumas vezes/Frequentemente
Item 9	No ano passado, jogar (a dinheiro) levou-te a discutir com a tua família/amigos/outros? A faltar recorrentemente à escola? Nunca/Uma ou duas vezes/Algumas vezes/Frequentemente

Instruções: As perguntas que se seguem dizem respeito a alguns comportamentos problemáticos de jogo a dinheiro. Pensa até que ponto estes comportamentos se podem aplicar a ti pensando no último ano. Procura responder a estas questões da forma mais honesta possível, uma vez que não há respostas certas ou erradas; estamos apenas interessados na tua opinião

Cotação: Os itens das respostas são cotados de acordo com as respostas fornecidas pelos participantes. Uma resposta “sim” no instrumento DSM-IV-MR-J nos itens 1 e 3 é representado pela resposta “frequentemente”. Uma resposta “sim” no item 2 é representada pela alternativa de resposta “sim”. Uma resposta “sim” nos itens 4 e 5 é representada por “algumas vezes” ou “frequentemente”. Uma resposta “sim” no item 6 é representada por “mais de metade das vezes” ou “sempre”. Uma resposta “sim” no itens 7, 8 e 9 é representada pelas alternativas de resposta “uma ou duas vezes”, “algumas vezes” ou “frequentemente”. Um participante que responda a quatro ou mais respostas “sim” é classificado como jogador problema
